# The Impact of Vasopressor and Sedative Agents on Cerebrovascular Reactivity and Compensatory Reserve in Traumatic Brain Injury: An Exploratory Analysis

**DOI:** 10.1089/neur.2020.0028

**Published:** 2020-11-06

**Authors:** Logan Froese, Joshua Dian, Carleen Batson, Alwyn Gomez, Norah Alarifi, Bertram Unger, Frederick A. Zeiler

**Affiliations:** ^1^ Biomedical Engineering, Faculty of Engineering, University of Manitoba, Winnipeg, Manitoba, Canada.; ^2^ Section of Neurosurgery, Department of Surgery, Rady Faculty of Health Sciences, University of Manitoba, Winnipeg, Manitoba, Canada.; ^3^ Department of Anatomy and Cell Science, Rady Faculty of Health Sciences, University of Manitoba, Winnipeg, Manitoba, Canada.; ^4^ Section of Critical Care, Department of Medicine, Rady Faculty of Health Sciences, University of Manitoba, Winnipeg, Manitoba, Canada.; ^5^ Centre on Aging, University of Manitoba, Winnipeg, Manitoba, Canada.; ^6^ Division of Anaesthesia, Department of Medicine, Addenbrooke's Hospital, University of Cambridge, Cambridge, United Kingdom.

**Keywords:** autoregulation, cerebrovascular reactivity, compensatory reserve, sedative drugs, vasopressors

## Abstract

The impact of vasopressor and sedative drugs on cerebrovascular reactivity in traumatic brain injury (TBI) remains unclear. The aim of this study was to evaluate the impact of changes of doses of commonly administered sedation (i.e., propofol, fentanyl, and ketamine) and vasopressor agents (i.e., norepinephrine [NE], phenylephrine [PE], and vasopressin[VSP]) on cerebrovascular reactivity and compensatory reserve in patients with moderate/severe TBI. Using the Winnipeg Acute TBI Database, we identified 38 patients with more than 1000 distinct changes of infusion rates and more than 500 h of paired drug infusion/physiology data. Cerebrovascular reactivity was assessed using pressure reactivity index (PRx) and cerebral compensatory reserve was assessed using RAP (the correlation [R] between pulse amplitude of intracranial pressure [ICP; A] and ICP [P]). We evaluated the data in two phases. First, we assessed the relationship between mean hourly dose of medication and its relation to both mean hourly index values, and time spent above a given index threshold. Second, we evaluated time-series data for each individual dose change per medication, assessing for a statistically significant change in PRx and RAP metrics. The results of the analysis confirmed that, overall, the mean hourly dose of sedative (propofol, fentanyl, and ketamine) and vasopressor (NE, PE, and VSP) agents does not impact hourly cerebrovascular reactivity or compensatory reserve measures. Similarly, incremental dose changes in these medications in general do not lead to significant changes in cerebrovascular reactivity or compensatory reserve. For propofol with incremental dose increases, in situations where PRx is intact (i.e., PRx <0 prior), a statistically significant increase in PRx was seen. However, this may not indicate deteriorating cerebrovascular reactivity as the final PRx (∼0.05) may still be considered to be intact cerebrovascular reactivity. As such, this finding with regards to propofol remains “weak.” This study indicates that commonly administered sedative and vasopressor agents with incremental dosing changes have no clinically significant influence on cerebrovascular reactivity or compensatory reserve in TBI. These results should be considered preliminary, requiring further investigation.

## Introduction

Vasopressors and sedative agents are one of the cornerstones of guideline-based intensive care unit (ICU) therapeutics for moderate/severe traumatic brain injury (TBI). Sedation agents including propofol, fentanyl, and ketamine are employed for the purpose of intracranial pressure (ICP) reduction, and suppression of cerebral metabolic demand.^1–3^ Similarly, vasopressor agents are employed to maintain cerebral perfusion pressure (CPP) guideline-based targets of 60–70 mm Hg,^3^ with commonly employed agents including norepinephrine (NE), phenylephrine (PE), and vasopressin (VSP). Although the systemic effects of these agents have been documented, a refined understanding of the cerebral responses to these agents is limited. Of particular interest, is the impact of such sedative and vasopressor agents on cerebrovascular reactivity and compensatory reserve in TBI.

Pressure reactivity (PRx) has emerged as a continuous measure of cerebrovascular reactivity,^4–6^ with impaired cerebrovascular reactivity associated with poor patient outcome in TBI.^7–12^ Cerebrovascular reactivity is emerging as an important component to ongoing cerebral physiological dysfunction in the setting of current guideline-based therapies.^13,14^ Further, impaired cerebrovascular reactivity has been demonstrated to be associated with computed tomography (CT) based peri-contusion edema progression.^15,16^ In addition, novel methods using cerebrovascular reactivity physiological targets, such as optimal cerebral perfusion pressure (CPPopt)^17–20^ or individual ICP (iICP) thresholds,^21,22^ have emerged in personalize treatment of patients with TBI. Similarly, continuously assessed cerebral compensatory reserve using the RAP index (correlation [R] between pulse amplitude of ICP [A] and ICP [P]),^23^ has been linked to CT evidence of diffuse intracranial injury^7^ and 6-month global outcomes in TBI populations.^24,25^

Currently, our understanding of the impact of guideline-based therapeutics, including the effects of sedatives and vasopressors, is limited to a small number of studies using aggregate data.^10,13,26,27^ A recent 25-year retrospective analysis by Donnelly and associates suggested that, despite changes in TBI management guidelines over various epochs, cerebrovascular reactivity remained essentially unchanged, with mortality rates in patients with moderate/severe TBI being relatively fixed.^10^ Similarly, a recent multi-center study from the Collaborative European NeuroTrauma Effectiveness Research in TBI (CENTER-TBI) study found no significant association between therapeutic intensity levels (TIL), and impaired cerebrovascular reactivity.^13^ Finally, a recent single-center study in neurocritically ill patients found that incremental changes in propofol and NE failed to elicit any significant responses in cerebrovascular reactivity.^27^ However, aside from this last study, the previous works were conducted using large aggregates of physiology data, with medication administration data that lacked temporal resolution, and relied on daily treatment measures.

Prior to widespread adoption and phase 3 evaluation of personalized physiological targets based on cerebrovascular reactivity and compensatory reserve monitoring, it is imperative that we understand the impact of commonly administered therapeutics on these metrics. As such, the goal of this study was to assess the influence that NE, PE, VSP, propofol, fentanyl, and ketamine have on cerebrovascular reactivity and compensatory reserve, using archived high-frequency physiology data and treatment information stored in the Winnipeg Acute TBI Database.

## Methods

### Study design

We retrospectively reviewed our prospectively maintained TBI database from the Winnipeg Acute TBI Laboratories, at the University of Manitoba. We selected those patients with archived high-frequency digital physiology (ICP and arterial blood pressure; ABP) and treatment data pertaining to vasopressor or sedative agent administration. All patients included in this database are age 17 years or older, who have suffered moderate to severe TBI, requiring admission to the surgical intensive care unit (SICU) for invasive ICP monitoring. Patients received treatment according to the Brain Trauma Foundation (BTF) guidelines.^3^ All patients were intubated and ventilated to maintain oxygen and carbon dioxide levels with arterial partial pressure of carbon dioxide falling within normal ranges (median: 37 mm Hg; interquartile range [IQR]: 35–43). A total of 38 patients were identified, and for each agent there were more than 30 infusion rate changes and more than 500 h of recorded cerebral data, with the exception of PE (3 distinct changes and 16 h of data). All aspects of data collection for this ongoing prospective TBI database, including patient demographics/treatment/outcome and high-frequency physiology, have been approved by the University of Manitoba Research Ethics Board (H2017:181 and H2017:188), with approval for retrospective access for this project (H2020:118). The need for informed consent has been waived by the Research Ethics Board for this study.

### Patient data collection

As part of the ongoing prospective TBI database, all patient demographic, injury, and treatment information is recorded. For the purpose of this study, we extracted standard patient demographics (including age, sex, admission Glasgow Coma Scale (GCS) total and motor scores, pupillary response, presence of pre-hospital hypoxia/hypotension, and admission CT characteristics, including Marshall CT scores. All drug infusion rates are recorded with a time stamp, allowing linkage to high-frequency recorded physiology.

All patients had ICP and ABP data prospectively recorded using Intensive Care Monitoring Plus (ICM+) software (Cambridge Enterprise Ltd., Cambridge, UK; http://icmplus.neurosurg.cam.ac.uk), with all signals recorded using the same software and digitized via an A/D converter (DT9804 or DT9826; Data Translation, Marlboro, MA, USA), where appropriate, sampled at a frequency of 100 Hz or higher. ICP was monitored using an intra-parenchymal strain gauge probe (Codman ICP Microsensor; Codman & Shurtleff Inc., Raynham, MA, USA). ABP was obtained through arterial lines connected to a pressure transducer. These methods are similar to those of prior works in the field.^10,14,28^

### Signal processing

The following signal processing occurred using similar methodology, covered in other publications by our group and the senior author.^10,14,28^ CPP was calculated as mean arterial pressure (MAP)-ICP. Pulse amplitude of ICP (AMP) was derived from the fundamental amplitude of the ICP waveform in the frequency domain, using Fourier analysis over a non-overlapping 10-sec moving window. A 10-sec moving window average filter was applied to the raw data to decimate the signals to 0.1 Hz, focusing on the frequency ranges associated with cerebral vasogenic activity.^20,29^ ICP, AMP, MAP (derived from ABP), and CPP were subsequently output into 10-sec by 10-sec comma separated value files.

Cerebrovascular reactivity was assessed through the derivation of the PRx. PRx was determined using standard means, by calculating the Pearson correlation coefficient between 30 consecutive 10-sec measures of ICP and MAP, updated every minute.^9^ Similarly, cerebral compensatory reserve was determined using the RAP index calculated in a similar fashion to PRx, but using AMP and ICP.^24^ Data for PRx and RAP were output into minute-by-minute resolution comma separated value files, for the analysis of the impact of systemic vasopressors on both cerebrovascular reactivity and cerebral compensatory reserve.

### Statistical analysis

All statistical analysis was performed using R statistical computing software (R Foundation for Statistical Computing (2020), Vienna, Austria, http://www.R-project.org/). Descriptive summary statistics for the patient population are provided in Table 1. Alpha for statistical significance was set at 0.05, with no correction for multiple comparisons given the exploratory nature of this work. Box plots, error-bar plots, and a locally estimated scatter plot smoothing (LOESS) plot were used to aid in the description of the data. The statistical analysis was split into two phases: (A) evaluation of mean hourly physiology, and (B) evaluation of physiology surrounding each dose change. This was conducted for the following medications: propofol, fentanyl, ketamine, NE, PE, and VSP.

**Table 1.** Patient Demographics and Clinical Characteristics for Entire Cohort

Characteristics	Number (%) or median (interquartile range)
*N* (patients)	38
Age (years)	43.5 (25.5-56.5)
Sex (male)	33 (86.8%)
Admission GCS	7 (5.25-8)
Admission GCS-Motor	5 (3-5)
Arterial partial pressure of carbon dioxide (mm Hg)	37 (35-43)
Pupillary light reflex	
Bilateral reactive	23 (60.5%)
Unilateral unreactive	6 (15.8%)
Bilateral unreactive	9 (23.9%)
Pre-hospital hypoxia	20 (52.6%)
Pre-hospital hypotension	3 (7.9%)
CT, epidural hematoma	6 (15.8%)
Mean ISS	25 (25-32)
AIS, head/brain	5 (4-5)
Marshall Classification Category of 1st head CT	
3	12 (31.6%)
4	9 (23.7%)
5	17 (44.7%)
Favorable GOS-E outcome 1 month	20 (52.6%)

Favorable GOS-E was defined as GOS-E score of 5–8.

AIS, Abbreviated Injury Scale; CT, computed tomography; GCS, Glasgow Coma Scale; GOS-E, Glasgow Outcome Scale-Extended; HTS, hypertonic saline; ISS, Injury Severity Score.

### Evaluation of mean hourly physiology

Initially all data were extracted for patients who received a given vasopressor or sedative agent. These data were separated into non-overlapping time windows; for our analysis a window size of 1 h was used (we tested windows ranging from 30 min to 8 h of data). If a window was missing more than 10% of its data it was discarded from the study. To compare these time windows, two plots were made: mean index value versus mean average infusion dose, and mean time of index over threshold versus mean average infusion dose.

The mean average infusion dose was found using the mean average infusion rate calculated over the time window (time window in shown examples was 1 h, with similar results occurring for all other time windows assessed). Next, the mean index value was calculated over each given time window. Last, the mean time of index over a threshold was found for each window. The following thresholds for PRx and RAP were used: (A) PRx above +0.3,^30,31^ and (B) RAP above +0.4.^7^ These thresholds were chosen as they have been quoted in the TBI literature to be associated with worse global outcome. In this way each window has a mean average infusion dose, mean index value for each cerebral response (PRx and RAP), and a mean time for index over threshold for each cerebral response.

For both comparisons, two techniques were used to demonstrate the data. First, a LOESS plot between the mean infusion dose and either the mean index value or mean time over threshold was created. The LOESS creates a trend line to help convey any response that the cerebral physiology may demonstrate between the different infused doses. Second, the infused doses were then binned from zero to maximum infused amount. At most, 11 equally segmented bins were used, with “no infused medication” being its own bin (i.e., 0, (0,1],(1,2],(2,3] …. (9,10]). For each of these bins an error bar was found by the mean and the 95% confidence interval for the whole bin. The error-bar plot and the LOESS plot for each comparison was then displayed on the same plot, giving two plots for every cerebral response and vasopressor or sedative agent.

Last, we repeated the aforementioned plots but we adjusted the data for case-mix of TBI severity for patients with decompressive craniotomy, patients undergoing only an evacuation of a hemorrhage, and patients who did not require an operation.

### Evaluation about each incremental dose change

For each infusion rate change of the vasopressor or sedative agent, a 4-h time window was extracted pre-/post-infusion rate change. Any time window that had insufficient data was discarded from the study. Next, two time windows of the same length of time were taken from the data, with one window taken immediately before infusion rate change and one taken post-infusion rate change plus a chosen delay. The window times compared varied from 5 to 60 min with a delay that varied from 5 to 180 min, the final window time and delay used for all the final evaluations in this analysis was a 30-min delay between two 30-min windows (this allowed all agents to reach full onset response). For each window, the time over the previously mentioned thresholds was found for each infusion rate change, then a box plot was derived for each time window. Next, a Mann-Whitney U test was performed between the two window data sets. Last, we compared the grand mean value across the time windows and a Mann-Whitney U test was preformed between the two mean value sets. The variations in data windows, and delays, failed to lead to any significant differences in the relationships described in the Results section. As such, we report only the details regarding analysis of 30-min data windows, pre- and post-dose change.

To analyze whether the cerebrovascular reactivity status prior to medication change impacted the physiological response seen, we further analyzed the impact of dose changes for those with “intact” PRx prior to change (i.e., 4 h mean PRx <0 prior to medication manipulation), and those with “impaired” PRx prior to change (i.e., 4 h mean PRx above +0.30 prior to medication manipulation). The 4-h window to calculate the mean PRx pre-medication manipulation was chosen to ensure the patient had sufficiently impaired or intact cerebral reactivity. Such a window length is consistent with that employed for optimal physiological target determination using PRx. For both the time over threshold and mean values, all of the agents were compared in the following eight groups: (A) increase in infusion rate with pre-change PRx >+0.3 and PRx <0, (B) decrease in infusion rate with pre-change PRx >+0.3 and PRx <0, (C) going from “no medication” to “on” medication with pre-start PRx >+0.3 and PRx <0, and (D) going from “on” medication to “off” medication with pre-stop PRx >+0.3 and PRx <0.

Last, as with the hourly physiology we repeated the aforementioned plots but we adjusted the data for case-mix of TBI severity for patients with decompressive craniotomy, patients undergoing only an evacuation of a hemorrhage, and patients who did not require an operation.

## Results

### Patient characteristics

Table 1 provides the core patient characteristics for all the 38 patients. The median age was 43.5 years (IQR: 25.5–56.5 years), with 33 patients being male. The number of dosing changes seen was: NE, 569; VSP, 79; propofol, 213; fentanyl, 138; and ketamine, 33. PE had a limited number of dose changes and recorded physiology/drug data, as such no further analysis was conducted on this medication. NE had an infusion rate ranging from 0 to 0.56 mcg/kg/min, VSP from 0 to 2.4 units/h, PE from 0 to 0.4 mcg/kg/min, propofol from 0 to 5 mg/kg/h, fentanyl from 0 to 400 mcg/h, and ketamine from 0 to 40mcg/kg/min. Of the 38 patients, 12 underwent a decompressive craniectomy, 10 underwent an operation involving only the evacuation of a hemorrhage, and 16 did not require cerebral operation.

### Vasopressor and sedative dose response: hourly data

**FIG. 1.**
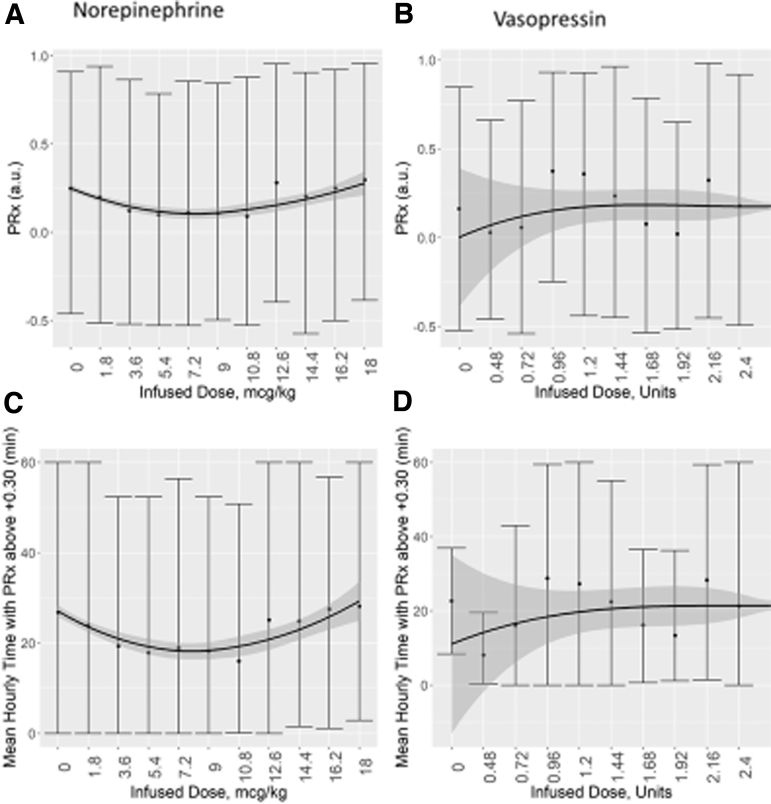
Mean hourly cerebrovascular reactivity versus mean hourly vasopressor dose: error-bar plots for NE and VSP. Top panels **(A,B)** demonstrate the mean hourly values for PRx versus mean hourly vasopressor dose infused. Bottom panels **(C,D)** demostrate the hourly time over PRx threshold of +0.30 versus mean hourly vasopressor dose infused. A/C panels are NE and B/D are VSP. A locally estimated scatter plot smoothing was performed and is indicated by the line with the 95% confidence intervals in the shaded area. The plots demonstrate no significant response of hourly PRx metrics to changes in NE or VSP dosing. a.u., arbitrary units; NE, norepinephrine; PRx, pressure reactivity index (correlation between intracranial pressure and mean arterial pressure); VSP, vasopressin.

**FIG. 2.**
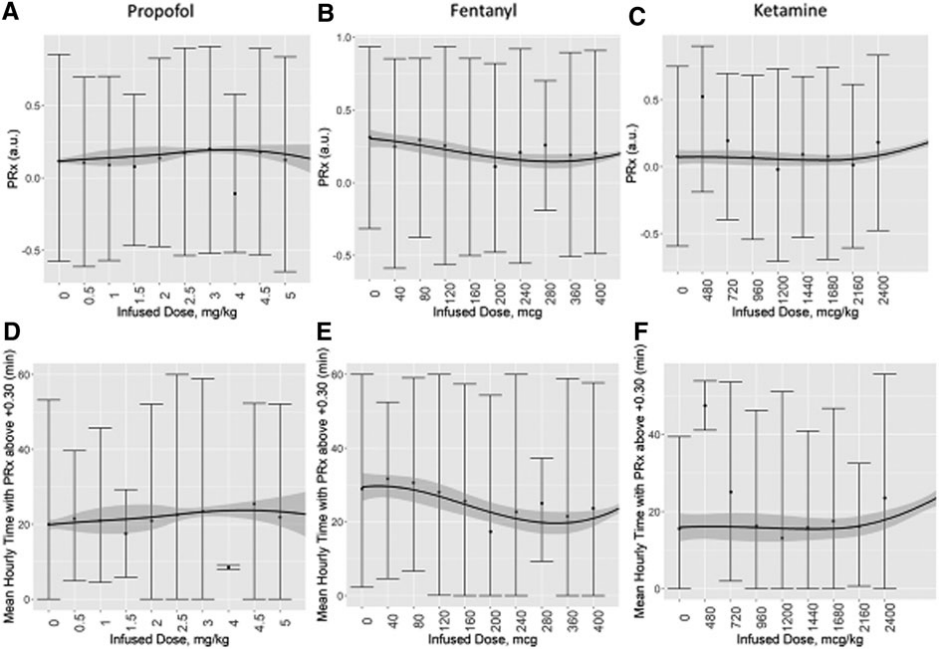
Mean hourly cerebrovascular reactivity versus mean hourly sedative dose: error-bar plots for propofol, fentanyl, and ketamine. Top panels **(A,B,C)** demonstrate the mean hourly values for PRx versus the mean hourly sedative dose infused. Bottom panels **(D,E,F)** demostrate the mean hourly time over PRx threshold of +0.30 versus the mean hourly sedative agent dose. A/D panels are propofol, B/E are fentanyl, and C/F are ketamine. A locally estimated scatter plot smoothing was performed and is indicated by the line with 95% confidence intervals represented by the shaded area. a.u. = arbitrary units; PRx, pressure reactivity index (correlation between intracranial pressure and mean arterial pressure).

Despite the wide variation of analyzed window times (30 min to 8 h) and the TBI severity of case-mix adjustment, none of the evaluated drugs had a significant specific influence on cerebrovascular reactivity or compensatory reserve, when evaluating the association between mean hourly physiology and mean hourly medication dosing. Supplementary Figure S1 provides results of the RAP analysis. Figure 1 demonstrates the vasopressors' influence and Figure 2 demonstrates the sedative agents. PE was not analyzed given the limited amount of data available in our data set (i.e., three distinct dose changes and 16 h of data, total). Of note, however, is the parabolic shape to the LOESS curve for the relationship between PRx and NE infused dose; this may be an indication of the CPP and PRx coupling effect seen with optimal CPP targeting.^17–20^

### Vasopressor and sedative incremental dose change response

**FIG. 3.**
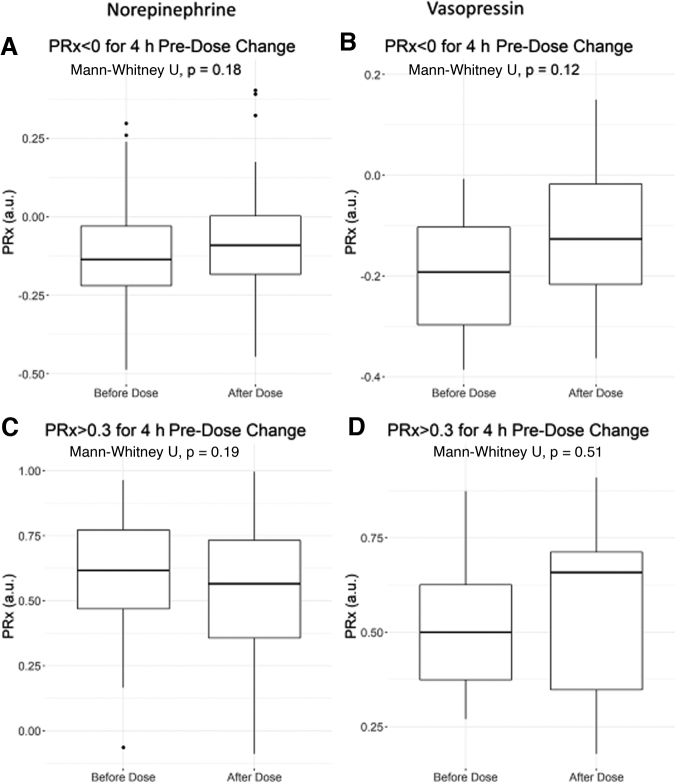
Mean PRx versus dose change in NE and VSP: box plot for increase in infusion rate. Top panels **(A,B)** demonstrate the mean PRx values pre-/post- vasopressor infusion increase for those instances where PRx was intact prior to dose change (i.e., PRx <0 for 4 h pre-dose change). The bottom panels **(C,D)** demostrate mean PRx values pre-/post-dose increase for instances where PRx was impaired prior to dose change (i.e., PRx >+0.3 for 4 h pre-dose change). The A/C panels are NE and B/D are VSP. A Mann-Whitney U test between each time segment was performed with *p*-values reported. a.u, arbitrary units; NE, norepinephrine; PRx, pressure reactivity index (correlation between intracranial pressure and mean arterial pressure); VSP, vasopressin.

**FIG. 4.**
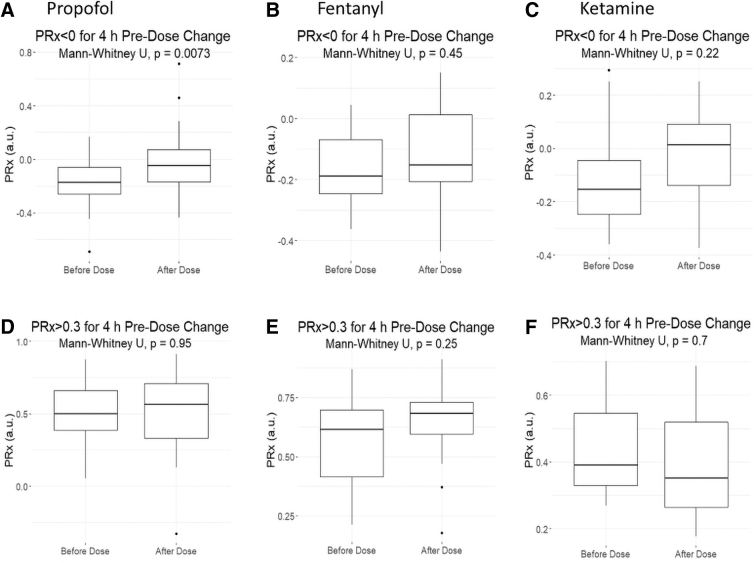
Mean PRx versus dose change in sedatives: box plots for increase in infusion rate. Top panels (**A,B,C**) demostrate mean PRx pre-/post-dose increase for instances where cerebrovascular reactivity was intact pre-dose change (i.e., PRx <0 for 4 h pre-dose change). The bottom panels **(D,E,F)** demostrate mean PRx pre-/post-dose increase for instances where cerebrovasular reacivity was impaired pre-dose change (i.e., PRx >+0.3 for 4 h pre-dose change). The A/D panels are propofol, B/E are fentanyl, and C/F are ketamine. A Mann-Whitney U test between each time segment was performed, with *p*-values reported. a.u., arbitrary units; PRx, pressure reactivity index.

In general, incremental dose changes in vasopressors and sedatives demonstrated little influence on PRx or RAP, regardless of the time periods of data compared pre- and post-dose change or the TBI severity of case-mix adjustment. Supplementary Fig. S1 provides the analysis results for RAP. For the purpose of uniformity in reporting, we focused on the comparison of 30 min of data, pre- and post-dose change with a 30-min delay. Figure 3 displays box plots of PRx and time with PRx above +0.30, pre- and post-dose change for NE and VSP. PE was not analyzed due to the limited data available. Figure 4 displays box plots of PRx metrics pre- and post-dose change for: propofol, fentanyl, and ketamine. The one exceptional response seen was with propofol.

An increase in propofol dose was associated with an increase in mean PRx and time spent with PRx above +0.30, if mean PRx pre-infusion was less than 0 (an indication of intact cerebrovascular reactivity; see Fig. 4A). This increase was statistically significant after 15 min of propofol infusion increase and remained for the 4-h analyzed window post-infusion rate increase. This trend was also seen in a similar scenario (i.e., PRx pre-infusion <0) with propofol infusion being started (off-to-on medication), and for each case of TBI severity (although only patients who did not require an operation reached an alpha significantly smaller then 0.05).

In all other groups evaluated (vasopressor agent, sedative agent, increase/decrease in dose, off-to-on medication, on-to-off medication, change in delay time, or change in window time) there was a non-significant response in PRx.

## Discussion

Using the unique temporally resolved high-resolution data set from the Winnipeg Acute TBI Database, we were able to preliminarily explore the relationships between various vasopressors/sedatives and cerebrovascular reactivity and compensatory reserve metrics. Evaluating both physiological responses to mean hourly dosing and each incremental medication dose change, some important aspects were highlighted.

First, cerebrovascular reactivity, as measured through PRx metrics, was not impacted by changes in mean hourly dose, or incremental dose changes, in vasopressor agents in this cohort. These agents included NE and VSP. We had insufficient data on PE to perform the analysis in our cohort. The findings regarding NE are corroborated by the recent publication by Klein and colleagues.^27^ This current work is the first such study to evaluate the cerebrovascular reactivity response to VSP. As these agents are commonly employed in guideline-based treatment of moderate/severe TBI, the lack of impact on cerebrovascular reactivity carries significance. It suggests that small incremental and daily dosing of NE and VSP do not need to be accounted for in future studies on cerebrovascular reactivity and individualized physiological targets derived from cerebrovascular reactivity. Although it must be acknowledged that this is only the second study to suggest this, and it does require further validation in larger multi-center data sets.

Second, the relationship between mean hourly NE dosing and PRx demonstrated a trend toward a parabolic relationship (as seen in Fig. 1). This finding may reflect the optimal relationship seen between PRx and CPP, with changes in NE driving changes in CPP. As such, variation in vasopressor dosing appears to provide some support for the ability to target optimal CPP targets. Such prospective work on optimal CPP targeting is the focus of collaborative groups in Europe and Canada,^12,27,32,33^ and the subject of an ongoing phase 2 randomized controlled trial.^22^

Third, cerebrovascular reactivity appears to remain relatively unaffected by sedative agents and changes in dosing. In particular, changes in fentanyl and ketamine failed to elicit significant alterations in PRx or time spent with PRx above +0.30. Of note, we did not have any data on benzodiazepine infusions to analyze. Propofol was the one exception, where in instances with intact cerebrovascular reactivity (i.e., PRx <0) pre-dose change, an increase in propofol dose led to a statistically significant increase in PRx, from a median of −0.174 (IQR: −0.258 to −0.061) to −0.048 (IQR: −0.171 to 0.072; *p* = 0.0073). This was based on 94 dose changes in propofol and was not seen in those with impaired cerebrovascular reactivity (i.e., PRx >+0.30) pre-dose change. It suggests that increasing propofol may lead to worse cerebrovascular reactivity in those with intact reactivity pre-dose change. However, it must be noted the increase in PRx seen was by ∼0.10 units, to a value of 0.05. The increase amount in PRx is of questionable significance, and a value of −0.05 is commonly considered intact cerebrovascular reactivity.^8,11,21^ As such, at this time, we cannot definitively say that propofol dose increases make a clinically significant change in PRx. The significant effect seen in our study, but not the prior work by Klein and colleagues,^27^ may just reflect the small sample size in our study. As well, propofol is vasoactive and causes decreases in systemic blood pressure; this could negatively impact CPP and may worsen PRx. Although the limited response of PRx to the other vasopressors may conflict with the idea that CPP manipulation alone is sufficient to demonstrate an alteration to PRx. These findings do, however, suggest a need for future investigation in to the impact of propofol on cerebrovascular reactivity, because if such findings are validated they may carry implications for personalized physiological targeting based on cerebrovascular reactivity monitoring.^18,21,22,34^

Finally, cerebral compensatory reserve, as assessed through RAP, did not appear to be impacted by changes in mean hourly dosing or incremental dose changes in either vasopressor agents or sedatives. This was the case for each of the medications evaluated. As RAP is a relatively new index in continuous bedside cerebral physiological monitoring in TBI, it is difficult to know how to interpret this. No other study has evaluated this index and its response to these agents. It is possible that such small incremental changes in sedation fail to change cerebral compliance significantly so that changes in RAP can be seen. On the other hand, it may be a failing of the index as a measure of cerebral compensatory reserve in TBI, as it has yet to be validated in experimental models to truly measure compliance. Although, it must be acknowledged that RAP is strongly associated with imaging characteristics of diffuse intracranial injury in TBI,^7^ and appears to reflect intracranial compliance in non-TBI cases, such as hydrocephalus.^23^ This uncertainty regarding RAP in TBI monitoring supports the need for further work on this index, its associations, and relationship to TBI guideline-based therapeutic interventions.

### Limitations

Frist, despite the large number of incremental dose changes and long periods of drug infusions, this study is based on a small patient cohort. As such, the described results should remain exploratory at this time, requiring much further validation using multi-center data sets. In particular, PE could not be analyzed properly, given the limited amount of data in this unique data set. Similarly, the ketamine analysis was based on a relatively small number of dose changes and length of medication infusion, and should be interpreted with caution. Further, we did not have any data on benzodiazepine infusions, a commonly administered sedation agent in TBI. Second, the focus on basic statistics and general descriptive analyses for the vasopressor and sedative agent response is quite broad and was the first natural step for this unique data set with temporally resolved physiology and treatment data. However, to interrogate individual dose-responses, future work in this area will require time-series techniques, valuating the multi-variate relationships during infusions. Finally, all described influences are influenced by systemic and individual patient characteristics that are not accounted for within this small exploratory study. As such, future validation studies in this area will require complex multi-variable modeling, accounting for individual patient characteristics, injury patterns, and co-administration of other guideline-based therapies.

### Future directions

As the above-described analysis is based on a relatively unique temporally resolved data set, much further future work is required in the area of the effect of vasopressors/sedation on continuously monitored cerebral physiology. First, this future work will require larger multi-center data sets, with both physiological and treatment data collected in relative high temporal frequency. Such work is the focus of ongoing collaborative research groups in moderate/severe TBI.^12,27,32,33^ Second, in addition to replicating the analysis provided above, future work would benefit from employing time-series modeling techniques pre- and post-dose change, to assess if there is a significant change in the multi-variate relationship. By employing Granger causality and vector autoregressive integrative moving average (VARIMA) techniques, with impulse response function analysis, further nuanced information regarding particular dose/medication effects on cerebral physiology may be uncovered. Third, subclass analysis for different dose-change responses is required. This work would benefit from machine learning approaches, including the application of latent class techniques. Finally, prospective intervention work in both animal models with TBI, and human patients with TBI are required. Such work could evaluate different bolus dose effects on cerebral physiology, employing multi-modal continuous cerebral physiological monitoring. All of the above future avenues of investigation are necessary and will inform us if such medication effects need to be taken into account during future monitoring studies, including those evaluating personalized physiological targets in TBI care.

## Conclusion

The results of the analysis confirmed that, overall, the mean hourly dose of sedative (propofol, fentanyl, and ketamine) and vasopressor (NE, PE, and VSP) agents do not impact hourly cerebrovascular reactivity or compensatory reserve measures. Similarly, incremental dose changes in these medications in general do not lead to significant changes in cerebrovascular reactivity or compensatory reserve. Overall, this study indicates that commonly administered sedative and vasopressor agents with incremental dosing changes have no clinically significant influence on cerebrovascular reactivity or compensatory reserve in TBI. These results should be considered preliminary, requiring further investigation using multi-center high-resolution data sets.

## Funding Information

This work was supported directly through the Manitoba Public Insurance (MPI) Neuroscience/TBI Research Endowment, the Health Sciences Centre Foundation Winnipeg, the Canada Foundation for Innovation (CFI; Project # 38583), Research Manitoba (Grant # 3906), the University of Manitoba – Department of Surgery Geographical Full Time (GFT) Research Grant, and the University of Manitoba Office of Research Services (ORS) – University Research Grant Program (URGP).

F.A.Z. receives research support from the MPI Neuroscience/TBI Research Endowment, the Health Sciences Centre Foundation Winnipeg, the United States National Institutes of Health (NIH) through the National Institute of Neurological Disorders and Stroke (NINDS Grant # R03NS114335-01), the Canadian Institutes of Health Research (CIHR Grant # 432061), CFI (Project # 38583), Research Manitoba (Grant # 3906), the University of Manitoba VPRI Research Investment Fund (RIF), the University of Manitoba Centre on Aging, and the University of Manitoba Rudy Falk Clinician-Scientist Professorship.

L.F. is supported through the University of Manitoba – Department of Surgery GFT Research Grant, and ORS –URGP.

C.B. is supported through the Centre on Aging Fellowship at the University of Manitoba, and A.G. is supported through the University of Manitoba Clinician Investigator Program.

## Supplementary Material

Supplemental data

## Abbreviations Used

ABP: arterial blood pressure
AIS: Abbreviated Injury Scale
AMP: pulse amplitude of intracranial pressure
BTF: Brain Trauma Foundation
CENTER-TBI: Collaborative European NeuroTrauma Effectiveness Research in Traumatic Brain Injury
CFI: Canada Foundation for Innovation
CIHR: Canadian Institutes of Health Research
CPP: cerebral perfusion pressure
CPPopt: optimal cerebral perfusion pressure
CT: computed tomography
GOS-E: Glasgow Outcome Scale-Extended
GCS: Glasgow Coma Scale
HTS: hypertonic saline
ICP: intracranial pressure
ICU: intensive care unit
iICP: individual ICP intracranial pressure
IQR: interquartile range
ISS: Injury Severity Score
LOESS: locally estimated scatter plot smoothing
MAP: mean arterial pressure
MPI: Manitoba Public Insurance
NE: norepinephrine
NIH: National Institutes of Health
NINDS: National Institute of Neurological Disorders and Stroke
ORS – URGP: University of Manitoba Office of Research Services – University Research Grant Program
PE: phenylephrine
PRx: pressure reactivity index
RAP: the correlation [R] between pulse amplitude of intracranial pressure [A] and intracranial pressure [P]
SICU: surgical intensive care unit
TBI: traumatic brain injury
TIL: therapeutic intensity levels
VARIMA: vector autoregressive integrative moving average
VSP: vasopressin
