# Supplementary figures and images for: The Impact of Vasopressor and Sedative Agents on Cerebrovascular Reactivity and Compensatory Reserve in Traumatic Brain Injury: An Exploratory Analysis
Source: Neurotrauma Rep. 2020 Nov 6;1(1):157–68. doi: 10.1089/neur.2020.0028 (PMC7703494; doi:10.1089/neur.2020.0028)

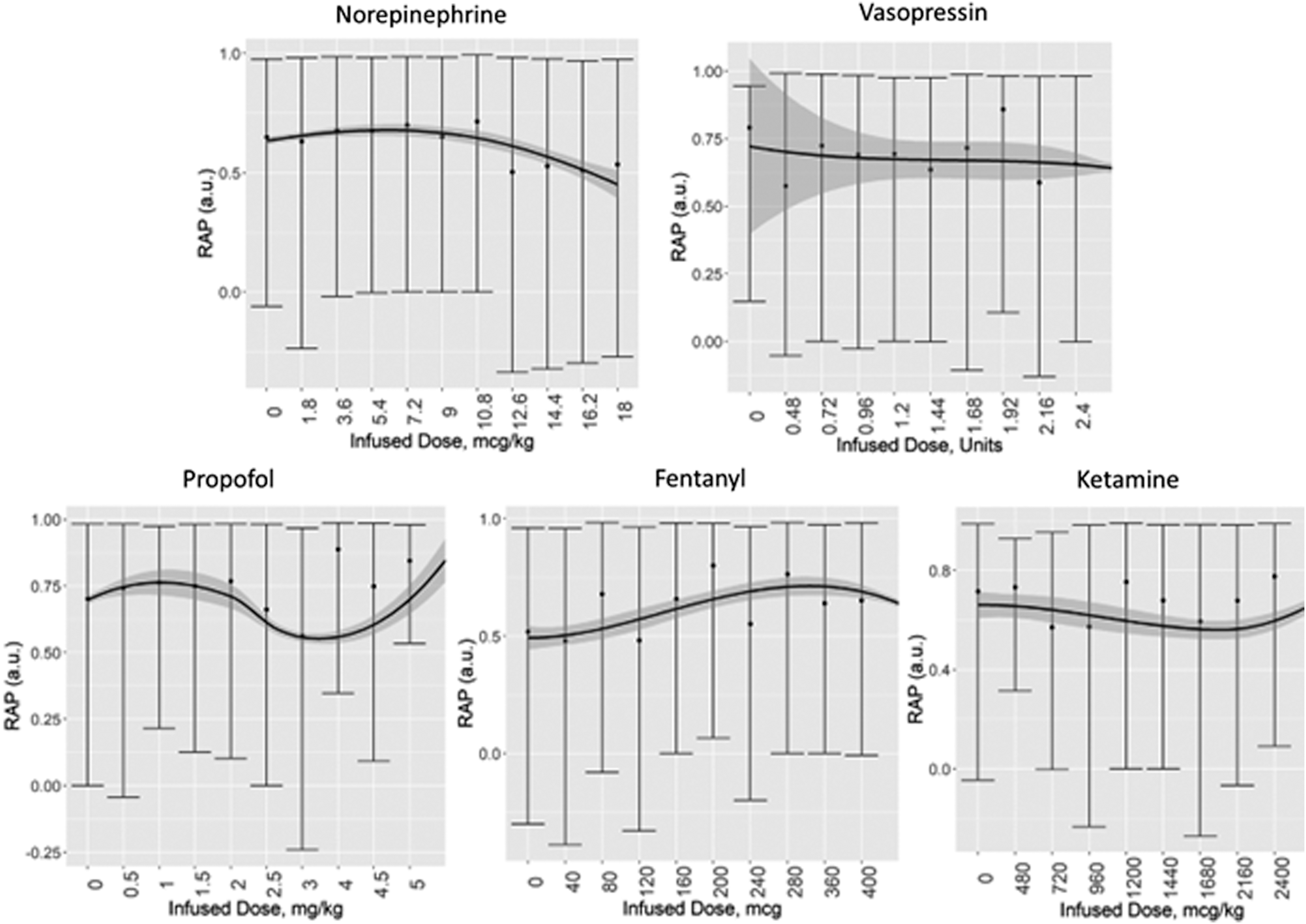

Supplement: Supplemental data [file Supp_Fig1.jpg]
